# Protective Effect of Mitochondria-Targeted Antioxidants against Inflammatory Response to Lipopolysaccharide Challenge: A Review

**DOI:** 10.3390/pharmaceutics13020144

**Published:** 2021-01-22

**Authors:** Ekaterina M. Fock, Rimma G. Parnova

**Affiliations:** Sechenov Institute of Evolutionary Physiology and Biochemistry of the Russian Academy of Sciences, Saint-Petersburg 194223, Russia; efock@mail.ru

**Keywords:** mitochondria-targeted antioxidants, inflammation, LPS, mitochondrial ROS

## Abstract

Lipopolysaccharide (LPS), the major component of the outer membrane of Gram-negative bacteria, is the most abundant proinflammatory agent. Considerable evidence indicates that LPS challenge inescapably causes oxidative stress and mitochondrial dysfunction, leading to cell and tissue damage. Increased mitochondrial reactive oxygen species (mtROS) generation triggered by LPS is known to play a key role in the progression of the inflammatory response. mtROS at excessive levels impair electron transport chain functioning, reduce the mitochondrial membrane potential, and initiate lipid peroxidation and oxidative damage of mitochondrial proteins and mtDNA. Over the past 20 years, a large number of mitochondria-targeted antioxidants (mito-AOX) of different structures that can accumulate inside mitochondria and scavenge free radicals have been synthesized. Their protective role based on the prevention of oxidative stress and the restoration of mitochondrial function has been demonstrated in a variety of common diseases and pathological states. This paper reviews the current data on the beneficial application of different mito-AOX in animal endotoxemia models, in either in vivo or in vitro experiments. The results presented in our review demonstrate the promising potential of approaches based on mito-AOX in the development of new treatment strategies against Gram-negative infections and LPS per se.

## 1. Introduction

Multiple diseases and pathological states caused by or associated with Gram-negative bacteria are accompanied by inflammation, local or systemic. Among them are respiratory and urogenital tract infections, endocarditis, gastritis, arthritis, meningitis, periodontal and endodontic diseases, diarrhea, and many other disorders [1,2]. Inflammatory processes that commonly accompany various noncommunicable diseases such as neurodegeneration, heart failure, cancer, diabetes mellitus, and others are exacerbated by the presence of Gram-negative infection. An extreme manifestation of inflammation is systemic inflammatory response syndrome, which is commonly observed in microbial infection-induced sepsis triggered by dysregulated inflammatory reactions and immunosuppression [3,4].

The most important virulent factor of Gram-negative bacteria that elicits the host’s innate immune response and causes acute inflammation is lipopolysaccharide (LPS), the major component of the outer membrane of bacteria. In eukaryotes, LPS is mainly recognized by membrane-bound Toll-like receptor 4 (TLR4) expressed in immune and other cell types. As an experimental tool, this endotoxin is widely used in both in vitro experiments on different cell types and in vivo animal models to mimic Gram-negative infection or sepsis. The development of effective therapeutic approaches against LPS is highly important. Even if bacteria are killed by antibiotics, the inflammation induced by LPS itself commonly remains clinically relevant [5]. Recent data have found that disruption of the paracellular intestine barrier due to gut dysbiosis can provoke the release of bacterial LPS into the systemic circulation, causing chronic, low-grade inflammation in different organs, including the brain [6,7,8]. This makes explorations with LPS particularly important, not only as a simulator of Gram-negative infection, but also as a toxic agent per se.

Considerable evidence indicates that oxidative stress and mitochondrial dysfunction are common features in the majority of inflammatory states and diseases, acute or chronic [9,10,11]. It is generally accepted that excessive generation of reactive oxygen/nitrogen species and mitochondrial injury driven by an uncontrolled inflammatory response play a central role in the genesis of multiple-organ failure observed in sepsis (for review, see [12,13,14,15,16]). Mitochondrial damage and imbalanced generation of reactive oxygen species (ROS) have been shown to contribute to local inflammatory pathologies induced by LPS of Gram-negative bacteria [17,18]. 

Mitochondria are a major source of cellular reactive oxygen species (ROS) and, at the same time, a vulnerable target of ROS damage. In addition, endogenous oxidant defense mechanisms that protect the cell from excess ROS generation can be overwhelmed in different pathological states. Impairment of mitochondria plays a critical role in inducing cell apoptosis and tissue damage in different inflammatory states. Evidence indicates that endotoxemia is accompanied by a significant elevation in mitochondrial ROS (mtROS) generation, impairment of the electron transfer chain (ETC) and oxygen consumption, reduction in the mitochondrial membrane potential (MMP), deficiency in ATP production, decline in the endogenous antioxidant capacity, and accumulation of lipid peroxidation products [19,20,21,22,23]. mtROS are also able to stimulate NADPH oxidase activity, enhancing the cytosolic ROS level [24]. Recent data have highlighted the role of LPS-triggered mtROS in NLRP3 inflammasome assembly on the surface of the mitochondrial outer membrane, leading to the maturation of proinflammatory cytokines [25,26]. Since a critical step in mitochondria disturbances is known to be associated with harmful ETC-mediated ROS generation [27,28,29,30,31], their scavenging by antioxidants delivered specifically to mitochondria can be beneficial for restoration of mitochondrial function, preventing the development of inflammation and tissue damage. Over the past 20 years, a large number of mitochondria-targeted antioxidants (mito-AOX) that can accumulate inside mitochondria and scavenge free radicals have been synthesized and tested in different in vitro and in vivo models (for review, see [9,32,33,34,35]). 

The most studied mito-AOX (MitoQ, SkQ, MitoTEMPO, and others) are conjugates of an antioxidant moiety with the triphenylphosphonium (TPP^+^) lipophilic cation, which enables the rapid uptake of the chimeric molecule within mitochondria due to the negatively charged membrane potential across the inner mitochondrial membrane [36,37,38]. Another class of mito-AOX is Szeto–Schiller (SS) tetrapeptides, which have antioxidant properties attributed to their tyrosine or dimethyltyrosine residue. They accumulate within IMM independently of the mitochondrial membrane potential, binding with high affinity to cardiolipin [39,40]. The protective role of these and other mito-AOX based on the prevention of oxidative stress and the restoration of mitochondrial function has been demonstrated in a variety of common diseases and pathological states, such as atherosclerosis, metabolic diseases, ischemia/reperfusion injury, hypertension, degenerative neurological disorders, aging, and others (for review, see [9,31,32,41,42])**.**

**Figure 1:**
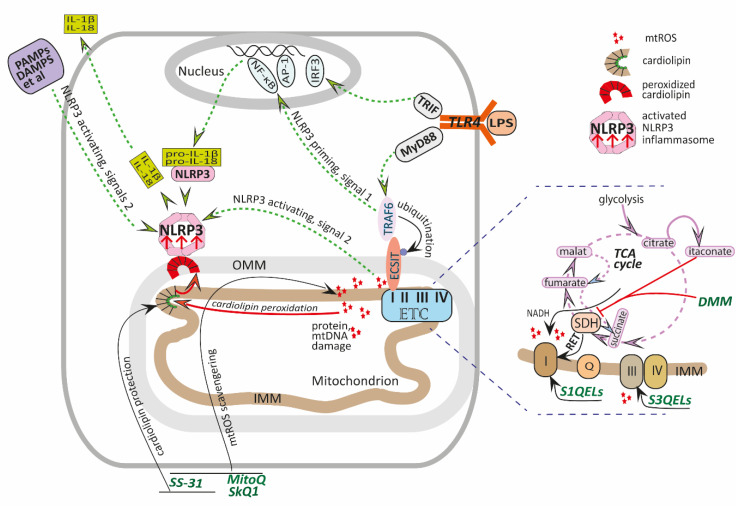
Diagram illustrating the protective mechanisms of mito-AOX against the LPS-induced cell inflammatory response. Activation of TLR4 by LPS triggers MyD88- or TRIF-dependent signaling pathways, resulting in the translocation of nuclear transcription factors NF-κB, AP-1, and IRF3 into the nucleus. This leads to the initiation of transcription of a wide range of pro- and anti-inflammatory mediators. LPS-induced priming of the NLRP3 inflammasome mediated by the adapter protein TRAF6 and NF-κB results in transcription of NLRP3 and inactive pro-IL-1β and pro-IL-18 proteins. In addition, TRAF6 is translocated to the mitochondria and ubiquitinates ECSIT, a protein implicated in complex I assembly and stability, resulting in its enrichment at the mitochondrial periphery and facilitation of mtROS production. Complexes I and III of the ETC are the main sites of mtROS production. In conditions of low forward electron transport, complex I can generate mtROS via NADH oxidation as well as via reverse electron transport (RET) from SDH to complex I (see the text). Inhibition of SDH by the TCA cycle derivative itaconate, as well as malonate or cell-permeable dimethyl malonate (DMM), attenuates RET and consequently diminishes mtROS production. mtROS serve as one of the numerous NLRP3 activating signals that cause the assembly and activation of the NLRP3 inflammasome complex and maturation and secretion of IL-1β and IL-18. In addition, mtROS damage proteins and mtDNA and induce lipid peroxidation. Oxidized cardiolipin is translocated from the IMM to the OMM, where it serves as a docking station for NLRP3. Mito-AOX, such as MitoQ, SkQ1, or SS-31, accumulate within the IMM to scavenge mtROS. SS-31, besides its ROS-scavenging properties, binds selectively to cardiolipin, increasing lipid packing of the membrane and tightening membrane curvatures, thus protecting the ETC and mitochondrial function. S1QELs and S3QELs, small molecules from different chemical families, specifically suppress mtROS production at ETC sites I_Q_ and III_Qo_, respectively. Abbreviations: DAMP—damage-associated molecular pattern; ECSIT—evolutionarily conserved signaling intermediate in Toll pathways; ETC—electron transport chain; LPS—lipopolysaccharide; IMM—inner mitochondrial membrane; NLRP3—NLR family pyrin domain containing receptor 3 inflammasome; OMM—outer mitochondrial membrane; PAMP—pathogen-associated molecular pattern; mtROS—mitochondrial reactive oxygen species; SDH—succinate dehydrogenase.

This paper reviews the current data on the beneficial application of different mito-AOX in animal endotoxemia models, in either in vivo or in vitro experiments. We focused mainly on LPS challenge, although the cecal ligation and puncture (CLP) model mimicking polymicrobial sepsis was also considered. The anti-inflammatory action of mito-AOX observed at the cellular or the tissue level in LPS-induced inflammatory models, as well as challenges concerning mito-AOX application and their therapeutic potential, was discussed. In addition, we summarized the current knowledge on the anti-inflammatory benefits of competitive inhibitors of succinate oxidation, such as itaconate and malonate, which indirectly reduce mtROS generation. The schematic representation of inflammatory pathways triggered by LPS and the mechanisms of mito-AOX protection is presented in Figure 1.

## 2. Mechanisms of LPS-Triggered Inflammation

Inflammatory reactions play a critical role in LPS-induced tissue injury, which is caused by the adhesion and migration of leucocytes through the epithelium, the production of a variety of proinflammatory mediators by monocytes/macrophages, and oxidative stress driven by excess generation of reactive oxygen and nitrogen species. As the most abundant proinflammatory agent, LPS activates the systemic and cellular inflammatory response largely due to signaling through Toll-like receptor 4 (TLR4), which is expressed not only in immune cells but also in almost all cell types [43,44,45]. In mammalian cells, LPS-induced activation of TLR4 occurs through a series of interactions with several adapter proteins to facilitate its recognition by the TLR4/MD-2 receptor complex, which causes TLR4 oligomerization and recruitment of its numerous downstream adaptors. LPS/TLR4 signaling can be divided into MyD88-dependent and MyD88-independent (TRIF-dependent) pathways, leading to nuclear transcription factors NF-κB-, AP-1-, and IRF3-mediated induction of a wide range of pro- and anti-inflammatory mediators, such as cytokines, chemokines, eicosanoids, and others [46,47]. Stimulation of immune, epithelial, endothelial, and other cells by pro-inflammatory mediators results in excessive ROS/RNS generation, which is potentially damaging to all cellular compartments. 

Recently, TLR4-independent LPS sensing pathways have been described [48]. First, transient receptor potential (TRP) channels have been identified as non-TLR membrane-bound sensors of LPS, and second, caspase-4/5 (and caspase-11 in mice) have been established as cytoplasmic sensors for LPS [48]. 

LPS has been shown to activate inflammasomes—cytosolic multiprotein complexes that mediate the propagation of the inflammatory response within the innate and the adaptive immune system as well as in epithelial cells [25,26,49,50]. Inflammasome activation is evoked by extra- and intracellular pathogens, such as Gram-negative bacteria, and/or by danger signals to activate caspase-1, resulting in a caspase-1-dependent, highly inflammatory form of cell death—pyroptosis [49,50]. It is thought that in Gram-negative infections, pyroptosis plays a key role in the destruction of intracellular bacterial replication niches. Inflammasomes are responsible for the maturation of proinflammatory cytokines, including IL-1β and IL-18, and for their secretion through the formation of pores in the plasma membrane [49,51,52]. The most studied inflammasome, NLRP3, is implicated in many different pathologies such as cancer and metabolic, neurodegenerative, and inflammatory diseases. Activation of the NLRP3 inflammasome occurs in two steps via different activating signals. Through TLR4 activation, LPS participates in the priming of inflammasome activation to initiate the transcription of NLRP3, inactive pro-IL-1β, and pro-IL-18 via NF-κB [51]. The second signals, which are notably diverse, include mtROS, derived mainly from ETC electron leakage [51], and initiate assembly of the inflammasome complex and, among other things, maturation of pro-IL-1β and pro-IL-18. 

## 3. Generation of mtROS and Their Role in Normal and Pathological Conditions 

ROS is a general term encompassing oxygen-free radicals, including superoxide ion (O_2_^−^) and hydroxyl radical (^·^OH), and nonradical oxygen substrates, e.g., hydrogen peroxide (H_2_O_2_) and singlet oxygen (^1^O_2_). ROS are produced during a wide range of biochemical reactions within the cell and within different cell compartments (mitochondria, peroxisomes, and endoplasmic reticulum). There are many cell proteins, bearing thiols, catecholamines, hydroquinones, and flavins, that may participate in intracellular ROS production. Although ROS can be produced in the cytosol by NADPH oxidases and xanthine oxidase, the major sources of ROS are mitochondria [30,31]. Simultaneously, mitochondria are a major target for damages by their own ROS.

Mitochondria can contain more than a dozen enzymatic sources of mtROS [53,54]. However, there is a consensus now that the mitochondrial respiratory chain is a primary source of mtROS, which are generated by the leakage of electrons from the ETC, resulting in a partial reduction in molecular oxygen. Mitochondrial complexes I (NADH-ubiquinone oxidoreductase) and III (cytochrome C oxidoreductase) are assumed to be a predominant source of superoxide generation under pathological conditions [30,55,56,57]. In particular, superoxide is generated by the reaction of O_2_ with electrons originating either from direct transport from NADH under conditions of a slow respiration rate (due to low ATP demand or mitochondrial damage) or from the reverse electron transport (RET) from complex II (succinate dehydrogenase) to complex I under conditions of a high protonmotive force or when there is a high NADH/NAD^+^ ratio in the matrix [30,55,56,57].

In healthy tissues under physiological conditions, mtROS generation and their removal by endogenous scavenging compounds and enzymes are tightly controlled by a complex antioxidant defense network including superoxide dismutase (SOD), catalase, ascorbic acid, tocopherol, reduced glutathione (GSH), etc. SOD catalyzes the conversion of superoxide to hydrogen peroxide, which, in turn, is converted to water by glutathione peroxidase (GPx). Under oxidative stress, the expression of antioxidant genes is activated by translocation of nuclear factor E2-related factor 2 (Nrf2) from the cytoplasm to the nucleus [11,58]. 

mtROS are necessary for the cell due to their essential role in various intracellular signaling pathways, including mitochondrial quality control by autophagy [59,60]. Disturbance of the fine-tuning of the equilibrium between mtROS generation and scavenging leads to excessive ROS production, resulting in severe damage of single cells and whole organs, loss of function, and then organism failure [61,62]. Numerous pathological conditions and diseases such as sepsis, cancer, metabolic diseases, neurodegenerative diseases, and others are triggered or accompanied by an increased level of mtROS [16,63,64]. At the cellular level, the most harmful effects of mtROS are associated with oxidation-triggered sustained damage of mitochondrial nucleic acids, proteins, and lipids, resulting in the impairment of ETC functioning and ATP production [64,65,66,67,68,69]. For example, mtROS-induced oxidation of cardiolipin polyunsaturated fatty acids damages cristae curvatures, reduces ETC complex activity, initiates cardiolipin translocation from the inner to the outer mitochondrial membrane, and triggers cytochrome C release into the cytosol, which is critical for the mitochondrial cell death pathway [70]. Furthermore, mtROS can oxidize proteins of the mitochondrial permeability transition pore, enhancing cytochrome C and mitochondrial DNA (mtDNA) release. mtDNA is especially sensitive to damage by ROS due to a lack of protective histones and its proximity to the ETC [71,72]. mtROS-initiated release of mtDNA is an important trigger of systemic inflammation, which is recognized by cells as a virulent pathogen motive [68]. Inflammatory conditions are commonly accompanied by excessive NO production, which, in turn, can interact with superoxide to generate the highly toxic peroxynitrite (ONOO^-^) [14,73]. 

## 4. LPS Triggers mtROS Generation

Enhancement of ROS generated in both the cytosol and the mitochondria is an important step in LPS signaling that links the activation of TLR4 with the NF-kB-driven expression of proinflammatory mediators. Numerous animal models of sepsis (LPS or CLP) have demonstrated common abnormalities: increased level of ROS, decreased antioxidant capacity, and mitochondrial oxidative damage [14,15,74]. LPS-triggered generation of mitochondrial superoxide measured usually with the fluorogenic dye MitoSOX was demonstrated in different cell types, such as microglia [75,76], muscle myoblasts [23], gingival fibroblasts [17], human pulmonary bronchial epithelial cells [77], macrophages [78], and others. In addition, the decline in antioxidative enzymes and glutathione content caused by LPS contributes to the impairment of endogenous antioxidant defense and the subsequent increase in mtROS generation [79,80,81,82,83]. 

In a wide range of cell types, LPS application disturbs cellular energetics, which manifests itself in a decline in respiratory complex activity, decline in the mitochondrial membrane potential, reduction in mitochondrial respiration, and suppression of ATP production in a tissue-, time-, and dose-dependent manner [19,22,28,84,85,86,87,88,89]. A critical step in these disturbances is associated with excessive ROS generation [27,28,29,30,31]. In innate immune cells, LPS has been shown to switch the metabolic reprogramming from oxidative phosphorylation to aerobic glycolysis as a survival response maintaining the cellular ATP level [75,78,90,91], which results in the slowing or reversing of electron transport through respiratory complex I and a subsequent increase in ROS production. 

The intrinsic mechanisms linking TLR4 signaling and mtROS have been studied mainly in phagocytic cells such as macrophages in which both cytosolic and mitochondrial ROS generation is also related to their bactericidal activity [92,93]. Thus, it has been shown that LPS-triggered induction of mtROS is mediated by a complex I-associated protein evolutionarily conserved signaling intermediate in Toll pathways (ECSIT), which plays a key role in complex I assembly and stability [93,94]. Upon LPS stimulation, the TLR signaling adapter tumor necrosis factor receptor-associated factor 6 (TRAF6) is translocated to the mitochondria and ubiquitinates ECSIT, resulting in its enrichment at the mitochondrial periphery, thus leading to the augmentation of mitochondrial and cellular ROS generation [93]. TRAF6 also mediates the ubiquitination of the small GTPase Rac, maintaining it in an active GTP-loaded state, which is necessary for the full activation of the ROS-producing machinery [95]. In addition, it was demonstrated in macrophages that LPS activates mitochondrial mitofusin2 (Mfn2) expression, which has been shown to be a required step for mtROS generation. *Mfn2^−^/^−^* macrophages are not be able to produce mtROS and proinflammatory mediators in response to LPS [96]. Whether the above mechanisms exist in other cell types remains unknown.

## 5. Mitochondria-Targeted Antioxidants and Their Application against LPS-Triggered Inflammation

Generally, antioxidants are substances that can accept or donate electron(s) to eliminate the unpaired condition of free radicals, thus neutralizing them. Antioxidant drugs can either scavenge free radicals or turn into new free radicals, which in some cases are less active and dangerous than initial ones [97]. Antioxidants can also break chain reactions [98] as well as affect ROS-regulated enzymes through controlling the cellular level of free radicals [99,100,101,102]. The natural defense mechanisms are supplied by enzymatic and non-enzymatic antioxidants, which are distributed within the cell cytoplasm and organelles (such as SOD, GPx and reductase, catalase, vitamins, minerals, polyphenols, albumin, transferrin, ferritin, and a variety of others), whereas foods and supplements provide a wide variety of exogenous natural ones (e.g., vitamins B, C, and E; ions Zn, Cu, and Se; flavonoids; omega-3 fatty acids; *L*-carnitine; and Q-enzyme Q10). 

### 5.1. Conjugates with Lipophilic Cations 

An era of investigations applying mito-AOX conjugates began half a century ago when V. Skulachev and colleagues demonstrated the ability of lipophilic cations, such as triphenylphosphonium (TPP^+^), to accumulate within mitochondria due to the large MMP (negative inside) [103]. In numerous chimeric mito-AOX compounds synthesized later, lipophilic cations, which provide drug accumulation several hundred-fold in the mitochondrial matrix, were grafted to antioxidant moieties, which quenched electrons from the respiratory chain, thus diminishing ROS elevation [104]. Conjugates designed in such a way are widely used as a tool for research, as well as for diagnostic and therapeutic purposes, including drug delivery (for review, see [105,106].

Triphenylphosphonium derivatives have been mainly used as a mitochondrial targeting moiety. They are conjugated with quinone derivatives (ubiquinone in MitoQs [36] or plastoquinone in SkQs [31,37]); with superoxide dismutase and catalase mimetics in MitoTEMPO and MitoTEMPOL, respectively [38]; and with vitamin E in MitoVitE [107], etc. Inside mitochondria, these chimeric mito-AOX undergo red/ox cycling: they not only quench radicals but also can be reduced afterward by the ETC. 

Most of the in vivo studies and clinical trials were performed with MitoQ and SkQ1. As both compounds are found to be localized at the matrix-facing side of the inner mitochondrial membrane with their antioxidant portion and alkyl chain, their main protective activity is to prevent lipid peroxidation [108,109]. TPP-based mito-AOX accumulate preferentially in healthy and hyperpolarized mitochondria but not within injured mitochondria, which carry a lower membrane potential and therefore are capable of taking up lower doses of therapeutic antioxidants than normal ones [110,111]. 

TPP-based antioxidants have been widely used in both in vitro experiments on various types of cells exposed to LPS (Table 1) and in vivo experiments on typical animal models of inflammation or sepsis (administration of LPS or cecal ligation and puncture; Table 2). Each cellular model mimics a specific pathological state or disorder associated with mtROS-induced inflammation. Among examples in Table 1 is LPS- or bacteria-induced mitochondrial dysfunction in oligodendrocytes (model of multiple sclerosis) [109], renal tubular cells (pyelonephritis and acute kidney injury) [18,112], microglia (neurodegeneration) [76], hepatocytes (liver failure) [113], endothelial cells (vascular abnormalities) [89,114], muscle myoblasts (diaphragm weakness) [23], gingival fibroblasts (periodontitis) [17], intestinal epithelial cells (impaired gut barrier function) [11], and others. 

The application of different TPP-based mito-AOX (MitoQ, MitoVitE, MitoTEMPO, or MitoTEMPOL) to primary cultured cells or to cell lines exposed to LPS convincingly evidences their antioxidant and mitochondria-protective properties. As shown in Table 1, TPP-based compounds commonly demonstrate a decrease in mitochondrial/cellular ROS generation, the enhancement of the content of GSH and antioxidant enzymes such as SOD and GPx, and decreased accumulation of lipid peroxidation products such as MDA, as well as restoration of mitochondrial function. These antioxidants decrease the production of proinflammatory cytokines such as IL-1β and IL-18 and prevent NF-kB and caspase activation, leading to the inhibition of apoptosis and the increase in cell survival. MitoTEMPO or MitoQ application highlights the critical role of mtROS in LPS/*E. coli*-induced inflammasome activation, as shown in colonic epithelial cells [120] and renal proximal tubular cells [112].

Examples of the beneficial application of TPP-based antioxidants in different murine and rat acute inflammation models are summarized in Table 2. TPP-based antioxidants have been shown to accumulate in all major animal organs, such as the heart, kidney, liver, lung, and others, after oral, i.v., or i.p. administration [104,129]. 

The heart and the cardiovascular system suffer seriously during sepsis. MitoQ administration largely prevents LPS-induced cardiac mitochondrial dysfunction and reduction in cardiac pressure-generating capacity, inhibiting caspase 9 and 3 activity [21]. The septic response is well known to be related to widespread vascular endothelial injury, which plays a key role in the progression of multiple-organ failure [130]. Results obtained on human endothelial cells (HUVECs) exposed to LPS+PepG showed that MitoQ decreases cellular ROS generation, restores the MMP, and attenuates pro-inflammatory mediator production [114] (Table 1). A protective effect of mito-AOX has been demonstrated in an animal model of acute kidney injury caused by CLP following MitoTEMPO i.p. injection six hours after operation [22] or by LPS administration following i.p. injection of SKQR1 (plastoquinol conjugated with decylrhodamine) three hours before LPS administration [121]. In both protocols, despite their differences, mito-AOX were nephroprotective (Table 2). SKQR1 was also highly protective against acute pyelonephritis induced by intraurethral infection [18]. In the frog urinary bladder epithelium, which possesses the characteristics of the mammalian kidney collecting duct, MitoQ effectively inhibited LPS-induced ROS generation, the decline in fatty acid oxidation, and subsequent accumulation of lipid droplets, demonstrating a key role of mtROS in the shift of intracellular lipid metabolism under the influence of bacterial stimuli [85]. 

The impairment of gut permeability is a serious consequence of dysbiosis. MitoQ has been shown to improve intestinal permeability and inhibit LPS-induced bacterial translocation via a decrease in oxidative stress and restoration of the level of tight junction proteins (occludin and ZO-1) in the gut epithelium [11]. The authors showed that MitoQ alleviates LPS-induced oxidative stress in intestinal epithelial cells, triggering the nuclear translocation of the nuclear factor Nrf2, which, in turn, stimulates the expression of its downstream antioxidant genes [11]. 

Data presented in Table 2 indicate that the protective effect of mito-AOX can be observed independently on the differences in the administration protocol (application of mito-AOX before, immediately after LPS administration /CLP or some time later). Even a six-hour delay in therapy with a single dose of MitoTEMPO significantly increased mitochondrial respiration and improved renal function and survival of animals [22]. Both immediate and delayed administration of the dismutase mimetic MitoTEMPOL was found to prevent sepsis-induced diaphragm weakness in a similar mode [74]. These observations are very important due to their clinical relevance. 

However, some studies have reported that TPP-conjugated compounds fail to inhibit mtROS-mediated injuries [131] or even have a detrimental effect on mitochondrial function. For example, in cultured mesangial cells, MitoQ, MitoTEMPOL, and MitoVitE at a dose of 1 µM inhibited oxidative phosphorylation [132]. Application of MitoQ (500 nM) to proximal tubule cells led to mitochondrial swelling and depolarization [133]. Both MitoQ (500 nM) and MitoTEMPOL (10 µM) had a marked negative effect on the respiration of myoblasts compared to controls [134]. The studies mentioned above revealed that the negative effect of TPP-conjugated compounds on mitochondrial function is related to the toxicity of the carbon alkyl chain of the cation moiety itself [132,133,134]. Another reason for TPP-conjugated mito-AOX toxicity is their ability to be pro-oxidants that generate superoxide via redox cycling [108,135]. A high concentration of antioxidants as well as other factors (the redox potential of matrix environments, the presence of Cu, Fe, and Zn ions) could reverse their behavior from anti- to pro-oxidant, subsequently causing toxic effects [111,136]. The pro-oxidant effect of MitoQ and other related compounds applied at high concentrations (more than 1 µM) has been shown to kill tumor cells, considering mito-AOX as potential chemotherapeutic drugs [111,137,138]. However, no pro-oxidant effect of MitoQ and other targeted quinones was demonstrated in mice who were fed antioxidants [139]. 

Since the probability of an adverse side effect of cation-conjugated mito-AOX provided by either a cation moiety or pro-oxidative behavior depends critically on their concentration; when dealing with this type of mito-AOX, it is particularly important to choose the relevant concentration, which, in turn, depends on a given cell type. For example, our experiments revealed that frog urinary bladder epithelial cells, demonstrating high tolerance to LPS, are very sensitive to the toxic effect of MitoQ (IC_50_ = 400 nM) [85]. At doses higher than 25 nM, it reduced the oxygen consumption rate and cell viability, whereas the antioxidant potency of MitoQ and the ability to restore the LPS-induced decline in fatty acids oxidation were observed at a dose of 25 nM [85], which is much less than that in most other in vitro works [92,140,141,142]. Of note, the concentrations of mito-AOX used in in vitro experiments were much higher than those that can be achieved pharmacologically and were associated with protective effects in vivo [9].

### 5.2. Other Mitochondria-Targeted Conjugates

There was an attempt to design mito-AOX using the mitochondrial protein import machinery, which delivers nuclear-encoded mitochondrial proteins inside the mitochondria via translocase through the outer and inner membranes (TOM and TIM complexes, respectively). A mitochondria-targeted macrocyclic SOD mimetic was synthesized by attaching the mitochondria-targeting sequence peptide to the porphyrin ring of the manganese porphyrin complex MnMPy4P. The resulting construct MnMPy3P–MTS reportedly demonstrated a decrease in LPS-induced cell death in activated macrophages [117]. 

Another example of the successful application of mito-AOX against LPS is the hemigramicidin–TEMPO conjugate XJB-5-131, which consists of a stable nitroxide radical and a portion of the membrane-active cyclopeptide antibiotic gramicidin S. The gramicidin segment was used to target the nitroxide payload to mitochondria because antibiotics of this type have a high affinity for bacterial membranes [119]. XJB-5-131 limited the LPS-induced inflammatory response both in vitro in macrophages and in vivo in a mice septic model [119].

### 5.3. Melatonin

Melatonin is a natural antioxidant produced mainly by the pineal gland as well as by most of the organs and tissues. Frequent use of melatonin for treatment of insomnia is based on its traditionally accepted role as a hormonal regulator of the circadian rhythm. Besides this, melatonin possesses antiapoptotic, anti-inflammatory, and antitumor activity, as well as powerful antioxidant properties. These facts alongside its profoundly safe side-effect profile make it possible to propose melatonin as a promising adjunctive drug for different pathological states, including inflammation and sepsis (for review, see [143,144,145,146,147]). 

Melatonin was first reported as a potent, broad-spectrum antioxidant and free-radical scavenger in the early 1990s [148]. The electron-rich melatonin molecule provides its antioxidant power via a cascade of scavenging reactions. Unlike classical antioxidants that have the potential to act as anti- and pro-oxidants via redox cycling [149], melatonin forms several stable end products excreted in the urine, which is believed to exclude its pro-oxidant effect [150]. Although the high lipid solubility of melatonin favors its entering all cells and subcellular compartments, melatonin is specifically targeted to mitochondria, where it enters via the oligopeptide transporters PEPT1 and PEPT2 [151]. In addition, melatonin is produced within mitochondria, and its generation can be inducible [152,153]. For these reasons, mitochondria have the highest level of melatonin. 

Melatonin is one of the most important endogenous factors in limiting oxidative stress. It provides antioxidant defense via a plethora of mechanisms. Melatonin by itself and also its endogenous metabolites directly scavenge free radicals, bind heavy metals associated with radical production, reduce the membrane potential, and stimulate ETC complex activity and ATP synthesis [154,155,156]. Moreover, melatonin potentiates the activity of a wide variety of antioxidant enzymes. It inhibits the ubiquitination of Nrf2, allowing its binding with the antioxidant response element, which, in turn, activates the transcription of antioxidant genes [157,158]. Melatonin augments the SIRT3 signaling pathway, which protects mitochondria from oxidative damage, upregulates the synthesis of GSH, and acts synergistically with vitamin C, vitamin E, and GSH to scavenge free radicals [149,159]. 

Numerous experimental studies have revealed the antioxidant and anti-inflammatory properties of melatonin, both in vitro and in vivo. Typical examples are presented in Table 1 and Table 2. On different cells challenged with LPS (HUVECs, cardiomyocytes, alveolar epithelial cells), it was shown that melatonin decreases ROS generation [79,81] and production of proinflammatory cytokines [80,81,116] and increases cellular antioxidant content (SOD, GSH) [79,80,115] through upregulation of Nrf2 expression [81]. Interestingly, not only melatonin but also its structurally related indolamine compounds (6-hydroxymelatonin, tryptamine or indole-3-carboxylic acid) possess antioxidant properties [80]. 

The beneficial application of melatonin was demonstrated in two animal models of sepsis—administration of LPS and CLP. Melatonin, being commonly injected i.p. before or after sepsis initiation, significantly improved sepsis-induced organ dysfunction (heart, kidney, liver, lung, placenta) by decreasing oxidative tissue damage and the inflammatory response, preserving mitochondrial function [79,83,116,122,123,160]. In the latest works on the septic cardiomyopathy model, it was shown that LPS suppresses the expression of B cell receptor-associated protein 31 (BAP31), a key regulator of endoplasmic reticulum stress, and melatonin could restore BAP31 expression. The knockdown of BAP31 attenuated the beneficial effects of melatonin on mitochondrial function and endoplasmic reticulum homeostasis under LPS [79], suggesting that, at least in part, melatonin contributes to the preservation of cardiac function in septic cardiomyopathy via regulation of BAP31 expression and stability. Another work demonstrated that autophagy plays a critical role in melatonin-induced myocardial protection. Thus, melatonin protects against LPS-induced septic myocardial injury by activating the AMPK-mediated autophagy pathway and further inhibiting mitochondrial injury and myocardial apoptosis [116].

### 5.4. Cell-Permeable Peptide Antioxidants

In the middle of the 2000s, a family of cell-permeable small synthetic tetrapeptides (Szeto–Schiller peptides (SS peptides)) was introduced as mitochondria-targeted antioxidants. The electron-scavenging abilities of SS peptides were provided by aromatic–cationic motifs in their molecules [39,161,162]. SS peptides readily penetrate the cell via diffusion, selectively accumulate within mitochondria, and concentrate in the IMM without reaching the mitochondrial matrix. In contrast to the MMP-driven entry of triphenylphosphonium-based conjugates into the mitochondria, the accumulation of SS peptides is independent of the MMP and does not depolarize the mitochondrial membrane. For this reason, SS peptides can penetrate not only normal mitochondria but also damaged ones with a low MMP [39]. 

The most studied peptide of this family is SS-31 (elamipretide, Bendavia™, MTP-131, d-Arg-Dmt-Lys-Phe-NH_2_), which, in addition to its mtROS-scavenging ability, links selectively to cardiolipin by electrostatic and hydrophobic interactions [40,163]. Thus, SS-31 is now positioned more as a cardiolipin stabilizer/protector than as a mtROS scavenger. 

Cardiolipin is readily oxidized by mtROS, which leads to multiple injuries. Oxidized cardiolipin disrupts the structure of respiratory supercomplexes to inhibit electron transfer and oxidative phosphorylation [70]. Translocation of oxidized cardiolipin from the IMM into the OMM provides a docking station for NLRP3 inflammasome assembly, and it can trigger mitochondrial fission and initiate mitophagy [164]. Binding of SS-31 to cardiolipin inhibits cardiolipin peroxidation, stabilizes cristae curvatures [40,163,165,166], and restores the stability and activity of respiratory complexes [167].

The linking of SS-31 to cardiolipin also inhibits the peroxidase activity of cytochrome C to result in decreasing mtROS production and improving the coupling between oxygen consumption and ATP synthesis [163]. SS-31 enhances ATP levels even under conditions of low substrate and oxygen supply, such as ischemia [40,165], or in increased energy demand states, such as sepsis and others pathologies [82,168,169]. The restoration of mitochondrial functioning by SS-31 can prevent a wide range of downstream cellular events, e.g., inflammasome activation and cytokine expression, autophagy, apoptosis, and necrosis. The beneficial effects of SS-31 were reported in different disease models (for review, see [32]), demonstrating the existence of a common mechanism mediating its action in different pathological conditions. 

The protective effect of SS-31 against LPS was demonstrated in several in vitro and in vivo models (see Table 1 and Table 2). In LPS-treated cells and CPL/LPS-challenged mice, SS-31 decreased apoptosis, improved sepsis-induced organ dysfunction, restored morphological damage, and reversed mitochondrial dysfunction [82,125,126,127]. It also attenuated ROS and MDA levels [82,125,126,127], maintained ATP production [82,126,127], and suppressed pro-inflammatory cytokine expression [82,125,126,127]. 

Several successive clinical trials in phases 1-3 were conducted in patients with cardiac, renal, skeletal muscle, and ophthalmic problems, as well as in mitochondrial myopathy patients (for review, see [32]). No adverse side effects of SS-31 were found until now. The safety of using SS-31, a drug with multiple beneficial pharmacological properties, for organs most affected by sepsis is particularly important. Very promising preclinical and clinical trial findings encourage to develop SS-31-based therapeutic approaches for the treatment of sepsis and other pathologies. 

### 5.5. Suppressors of Site IQ and IIIQ Electron Leakage

Recently, small molecules from different chemical families that specifically suppress mitochondrial superoxide/H_2_O_2_ production (S1QELs for site I_Q_ [170] and S3QELs for site III_Qo_ [171]) were identified by chemical screening. They bind directly to complex I or III and selectively suppress electron leakage without inhibiting oxidative phosphorylation [170,171], as well as inhibit the reverse electron flow through complex I [172]. They do not cause cytotoxicity at their effective concentrations [171] and do not participate in redox recycling [173]. 

The cytoprotective effect of S1QELs against oxidative damage has been demonstrated in animal (rat, mouse), human, and different cellular models [171,174,175]. S1QELs protected against ischemia-reperfusion injury in a perfused mouse heart [176]. In a murine model of asystolic cardiac arrest, S1QELs diminished myocardial ROS, as well as improved myocardial function after cardiopulmonary resuscitation, neurologic outcomes, and survival [177]. In recent papers, S1QELs and S3QELs have been offered as promising investigation tools for elucidating the functioning of I_Q_ and III_Qo_ sites in normal and pathological conditions, opening up new possibilities for better therapy [173,178]. Given the fact that LPS-driven mtROS are generated predominantly by mitochondrial complex I, S1QELs can potentially be specific suppressors of LPS-induced mtROS production, gently withstanding LPS-induced oxidative stress. However, the efficiency of S1QELs and S3QELs in a sepsis animal model or LPS-induced injury remains poorly investigated and warrants further research. 

## 6. Indirect Control of mtROS by Competitive Inhibitors of Succinate Dehydrogenase (SDH)

The accumulation of the citric acid cycle intermediate succinate, tightly connected with mtROS generation, has been shown to be a common cellular response to different pathological challenges such as ischemia/reperfusion, cancer, and inflammation [179,180,181,182]. An increase in the succinate level arises from SDH, operating in its opposite direction, which, in turn, is driven by fumarate overflow from purine nucleotide breakdown and partial reversal of the malate/aspartate shuttle [182]. Significant LPS-induced succinate accumulation was observed in macrophages [183], in which SDH activity is critical for determining the inflammatory phenotype of macrophages [78]. Subsequent rapid oxidation of succinate to fumarate by SDH under a large proton-motive force fuels RET, resulting in substantial generation of mtROS [78,179,180], which enhances pro-inflammatory cytokine expression by stabilizing hypoxia-inducible factor 1-alpha (HIF-1α) and suppresses the production of anti-inflammatory factors [78,183,184]. 

This pro-inflammatory scenario and metabolic reprogramming of immune cells are switched off by the generation of itaconate, another derivate of the citric acid cycle [185,186]. Itaconate produced from cis-aconitate is one of the most highly induced metabolites in LPS-activated macrophages, being an endogenous SDH inhibitor [78,185,186]. A significant decrease in LPS-stimulated mtROS and ROS-mediated cell damage was demonstrated in bone marrow-derived macrophages in the presence of 4-octyl itaconate or dimethyl itaconate, cell-permeable derivatives of itaconate [185,187]. In addition, in the cytosol, itaconate promotes the expression of anti-inflammatory and antioxidant genes by modifying the protein KEAP1, resulting in nuclear factor Nrf2 activation [188], as well as through the induction of an anti-inflammatory IkappaBzeta/ATF3 axis [186] to inhibit inflammasome activation. Thus, exposure to LPS not only promotes pro-inflammatory signaling via the succinate/mtROS pathway but also triggers a negative-feedback loop through itaconate-mediated induction of an anti-inflammatory program by SDH inhibition, as well as transcriptional factor Nrf2 and IkappaBzet/ATF3 activation. Dimethyl itaconate and 4-octyl itaconate were protective against LPS-induced injury in vivo [187,189]. Nevertheless, no substantial de-esterification of ester derivatives of itaconate was observed in activated macrophages, and only itaconate, but not its ester derivatives, led to increased intracellular succinate accumulation [189]. Further research is required to clarify this contradiction. 

Another potent endogenous competitive inhibitor of SDH, malonate, also acts as an indirect mitochondrial antioxidant by inhibiting succinate-driven RET. Dimethyl malonate (DMM), a cell-permeable malonate derivative, which is rapidly hydrolyzed in the cell to generate malonate, was used in both in vitro and in vivo models as an indirect mitochondria-targeted antioxidant [78,190]. In bone marrow-derived macrophages treated by LPS+succinate, DMM increased basal and LPS-induced cytosolic succinate levels and decreased the production of cellular ROS and proinflammatory cytokines. Mice treated i.p. with DMM before stimulation with LPS demonstrated a decrease in serum IL-1β and an increase in IL-10 [78]. The potential anti-inflammatory benefits of DMM were investigated in a mouse model with LPS/d-galactosamine-induced acute hepatic damage. DMM significantly alleviated hepatic damage and systemic inflammation [190]. In macrophages, it was also found that DMM suppresses the expression of gene sets associated with inflammation, including IL-1β and other HIF-1α-dependent genes, wherein many genes that were upregulated by succinate were reciprocally downregulated by DMM [78,190].

## 7. Conclusions

In recent years, the worldwide spread of Gram-negative infections, both chronic and acute up to sepsis, common and nosocomial, continues to pose a threat to human health. The abundance of Gram-negative infections is closely related to the overuse of antibiotics, immunosuppressive therapy applied in cancer, organ transplantation, heart surgery, etc., as well as to the prevalence of invasive devices and procedures, opening the gate for infection [191]. The most serious problem of recent decades has become the failure of conventional antibiotics to fight against multidrug-resistant Gram-negative bacteria. Moreover, LPS, the endotoxin of Gram-negative bacteria, inevitably triggers the host’s innate immune response and acute inflammation regardless of whether it remains in the membrane of alive or dead bacteria or is in cell-free form. The potential contribution of LPS and other toxins secreted by the gastrointestinal tract microbiome to human inflammatory disease is becoming increasingly acknowledged [6]. Thus, it has been shown that a particularly pro-inflammatory LPS subtype from the intestinal microbiome can penetrate the systemic circulation, cross the blood–brain barrier, and accumulate within CNS neurons, complicating or accelerating the development of neurodegenerative disorders such as Alzheimer’s disease [7]. In this connection, the development of new therapeutic approaches directed to the resolution of LPS-driven inflammation and to the improvement of the outcome of the pathology is very important. For this reason, the introduction of anti-inflammatory strategies employing mito-AOX into clinical practice promises to be especially attractive. 

The development of mito-AOX was based on approaches that allow one to limit excessive ROS production inside mitochondria via different mechanisms. Up to now, our knowledge of mtROS generation, their role in cell signaling and their impact on cellular antioxidant and pro- and anti-inflammatory mechanisms, is still insufficient and scant [10]. Nevertheless, besides the beneficial application of mito-AOX in numerous animal pathogenic models mentioned above, many clinical studies demonstrated the protective efficacy of mito-AOX in different pathological conditions wherever inflammation as well as mitochondria damage are involved. Thus, several successive SS-31 clinical trials in phases 1–3 were conducted in patients with cardiac, renal, skeletal muscle, and ophthalmic problems, as well as in mitochondrial myopathy patients (for review, see [32]). The safety of melatonin, which is widely used for counteracting sleep disturbances, was confirmed in a set of trials (for example, see Identifier: ChiCTR-TRC-13003997, ISRCTN15529655). Meta-analysis of randomized controlled trials demonstrated the effectiveness of melatonin in suppression of oxidative stress, which accompanies different pathological states [192]. In addition, melatonin was effective in newborns as an adjunctive therapy for sepsis [193] as well as in patients with *H. pylori*-associated dyspepsia [194]. 

MitoQ is now ubiquitously available as a dietary supplement. In clinical trials, it has shown efficiency in improving vascular function in middle-aged and elderly people and significantly decreased liver enzymes raised due to hepatitis C. Although it had no effect on Parkinson’s disease progression, no adverse side effects of MitoQ have been observed when it was daily administered to patients for a year [195]. SkQ1-based Visomitin eye drops were approved for clinical use in Russia, and their safety and efficacy were confirmed in phase 2 US clinical trials. Interestingly, the direct suppression activity of SkQ1 at micromolar concentrations toward the growth of different Gram-positive and Gram-negative bacteria has been found recently [196], suggesting that SkQ1 lowering the bacterial membrane potential may also be effective in the protection of infected mammalian organs by killing invading bacteria. In recent years, exploration of the development of SDH inhibitors and clarification of the intrinsic mechanisms of their action has been also intensified, promising novel therapeutic strategies to limit inflammation. 

Despite successful clinical trials, as mentioned above, the development of drugs based on mito-AOX and their application to Gram-negative infection are still in their infancy. Although numerous in vitro and in vivo studies have clearly demonstrated the protective effects of mito-AOX in different infection models, these results have not been translated to the clinic up to now. Future studies are needed to elucidate the time dependence and especially the long-term impact of mito-AOX application during chronic infection. Nevertheless, we believe that mtROS-targeted approaches possess great treatment potential and are worthy of being incorporated into preventive and therapeutic strategies against inflammation driven by Gram-negative infection. More research efforts are needed in the future to achieve this goal.

## Figures and Tables

**Table 1:** In vitro effects of mitochondria-targeted antioxidants.

Inflammatory Model	Cells	Mito-AOX	Major Findings	Reference
LPS *E. coli*	Macrophages	MitoQ 50-100 nM	↓ Cellular ROS; ↓ IL-1β mRNA and protein expression	[92]
LPS *E. coli*	BV-2 murine microglial cells	MitoTEMPO 200 µM	↓ Mitochondrial and cellular ROS; ↓ iNOS and COX-2 expression; ↓ TNF-α, IL-1β, IL-6 content; ↓ NF-ḵB activation	[76]
LPS *E. coli*	Primary cultured frog urinary bladder epithelial cells	MitoQ 25 nM	↓ Cellular ROS; prevention of fatty acid oxidation decline and lipid droplet accumulation	[85]
LPS *E. coli*	Intestinal epithelial cell line-6 (IEC-6)	MitoQ 1 µM	Stimulation of nuclear translocation of Nrf2	[11]
LPS *E. coli*	NRK-52E (rat renal proximal tubular cell line)	MitoQ 1 µM	↓ Cellular ROS; ↓NLRP3 inflammasome activation; ↓ IL-1β, IL-18, and caspase-1	[112]
Mixture of cytokines + LPS *E. coli*	C2C12 muscle myoblasts	MitoTEMPOL 10 mg/L	↓ Mitochondrial superoxide generation; prevention of reduction in cell width	[23]
LPS *E. coli +* PepG *S. aureus*	HUVEC-C	MitoQ 1 µM	↓ Cellular ROS; restoration of MMP; ↓ IL-1β, IL-6, and IL-8	[114]
LPS *E. coli +* PepG *S. aureus*	HUVEC-C	Melatonin, 0.1, 1.0, 10, 100, and 500 µM; MitoVitE, 5 µM	↓ IL-6 and IL-8; ↓ NF-ḵB activation; ↓ loss of MMP; ↑ GSH level; ↓ decline in metabolic activity	[80,115]
LPS *P. gingivalis*	Human gingival fibroblasts	MitoTEMPO 50 µM	↓ Mitochondrial ROS; ↓ IL-6, IL-1β, and TNF-α production; ↓ activation of NF-B	[17]
Inflammatory mediators generated by incubation of white blood cells with LPS *E. coli*	Buffalo rat liver cell line-3A (BRL-3A)	MitoTEMPO, 500 nM	↓ Mitochondrial and cellular ROS; ↓iNOS mRNA; ↓ IL-6	[113]
LPS *E. coli* + IFN-γ + TNFα	HUVEC	Mitoquinone (MQ) 1 µM	↓ Cellular ROS; ↓ tyrosine nitration and iNOS protein expression; recovery of O_2_ consumption and complex I activity	[89]
LPS *E. coli* + succinate	Bone marrow-derived macrophages	Dimethyl malonate, 10 mM; MitoQ, 500 nM; MitoTEMPO, 0.5–1 mM	↓ Cellular ROS, IL-1β, and HIF-1α; ↑ IL-1RA and IL-10	[78]
*E. coli* lysate + activated leucocytes	Primary culture rat kidney cells	SKQR1 10 nM	↓ Cellular ROS; ↓ cell dearth	[18]
LPS *E. coli*	Primary oligodendrocytes	SkQ1 5-10 nM	Restoration of myelin synthesis	[109]
LPS *E. coli*	Cardiomyocytes	Melatonin 100 μM	↓ Cellular ROS; ↓ loss of MMP; ↑ content of GSH, SOD; ↓ decline in BAP31 expression; ↑ cell viability	[79]
LPS *E. coli*	Primary neonatal rat cardiomyocytes	Melatonin 100 μM	↓ IL-6, TNF-α, mRNA levels; ↓Bax and ↑ Bcl-2 expression; ↑ autophagy;	[116]
LPS *E. coli*	Human alveolar epithelial cells	Melatonin, 800 μM	↓ cellular ROS; ↓ MDA; ↑ SOD and GPx levels; prevention of LPS-induced epithelial–mesenchymal transition through Nrf2 activation	[81]
LPS *E. coli*	Macrophages RAW 264.7	Mn-porphyrin-oligopeptide conjugate, 10 µM	↓ LPS-induced cell dearth	[117]
LPS *E. coli*	Cardiomyocytes (H9C2 cell line)	SS-31, 10 μM	↓ ROS; ↓ MDA; ↓ mRNA level of IL-6, IL-1β, and TNF-α; normalized activity of GPx and SOD; ↓ MMP decline; ↑ ATP	[82]
LPS *E. coli*	Murine microglial cells (BV-2)	SS-31, 100 nM	↓ ROS; effect is mediated by Fis1; ↓ Fis1 expression; ↓ COX-2 and iNOS expression	[118]
LPS *E. coli*	Macrophages RAW 264.7	XJB-5-131 2 µmol/kg	↓ NO and inflammatory cytokines	[119]
*E. coli* 0157:H7	Human colonic epithelial cell line (Caco-2)	MitoTEMPO	↓ Cellular ROS; ↓NLRP3 inflammasome activation; ↓ IL-1β and IL-18	[120]

**Table 2:** In vivo effects of mitochondria-targeted antioxidants.

Model of Infection	Species	Organ Investigated	Mito-AOX	Mode of Antioxidant Application	Major Findings	Reference
LPS *E. coli*	Rat, mouse	Heart	MitoQ 500 μM	Given water orally for 2 days	↓ Oxidative stress; ↓ mitochondrial dysfunction; ↓ cardiac TNF-α level; ↓ reductions in cardiac pressure generation; ↓ caspase 3 and 9 activity	[21]
LPS *E. coli*	Mouse	Gut, serum	MitoQ 4 mg/kg	i.v. injection 15 min before LPS	↓ Gut barrier dysfunction, restoration of the level of tight junction proteins (ZO-1 and occludin); ↓ intestinal inflammatory response; ↑ SOD and GSH level; ↓TNF-*α*, IL-1, IL-6, and NO in intestines and plasma	[11]
LPS *E. coli*	Rat	Liver, serum	MitoTEMPO 50 nmol/kg; SKQ1 5 nmol/kg	i.p., 24 and 1 h before LPS	↓ iNOS expression; ↓ plasma NO; ↓liver damage	[113]
LPS *E. coli*	Rat (7-day-old pups)	Kidney	SkQR1 100 nmol/kg	i.p., 3 h before LPS	↓Acute kidney injury; preservation of cell proliferative activity	[121]
*E. coli* lysate	Rat	Kidney	SkQR1 500 nmol/kg in total	i.p., 1, 12, 24, 36, and 48 h after intraurethral bacteria injection	↓ Renal cell dearth and animal mortality, restoration of Bcl-2 level in kidney; ↓TNF-α in kidney	[18]
LPS *E. coli +* PepG *S. aureus*	Rat	Liver, kidney, lungs, heart, gut	7.5 μmol/kg MitoQ, then 5 μmol/kg/h MitoQ	As a bolus i.v. infusion for 6 h after LPS+PepG	↓ Acute liver and renal dysfunction; ↑ MMP in most organs	[114]
LPS *E. coli* + PepG *S. aureus*	Rat	Liver, kidney	1.5 μmol/kg MitoQ or MitoVitE or melatonin, then 1 μmol/kg/h MitoQ, MitoVitE, or melatonin	As a bolus i.v. infusion for 5 h after LPS+PepG	↓ Mitochondrial damage; ↓ organ dysfunction; ↓ inflammatory response	[122]
CLP	Mouse	Kidney	MitoTEMPO 10 mg/kg	i.p., 6 h after operation	↓ Mitochondrial ROS, protection of complex I and II/III respiration; ↑ SOD; ↓ renal dysfunction (improved renal microcirculation and GFR); ↑ survival of animals	[22]
CLP	Mouse	Diaphragm	MitoTEMPOL 10 mg/kg/d	i.p., immediately after operation and 24 h later or only 6 h after operation	↓ Diaphragm weakness; ↓ mitochondrial superoxide generation; prevention of mitochondrial dysfunction; ↓ proteolytic enzyme activities; ↓ depletion of myosin heavy-chain protein content	[23]
LPS *E. coli*	Mouse	Heart	Melatonin 20 mg/kg/d	i.p., 48 h before LPS	↓ Cardiomyopathy; ↓ caspase 3 activation and cardiomyocyte apoptosis	[79]
LPS *E. coli*	Mouse	Heart	Melatonin 20 mg/kg/d	i.p., for 7 days before LPS	↓ Myocardial dysfunction and inflammation; ↓ cardiomyocyte apoptosis; ↑ AMPK activity and autophagy	[116]
LPS *E. coli*	Mouse (pregnant)	Placenta	Melatonin 5.0 mg/kg	i.p., 30 min before and 150 min after LPS	↓ Placental oxidative stress, hypoxic stress, and ER stress	[123]
CLP	Mouse	Diaphragm	Melatonin 30 mg/kg	i.p., four doses: 30 min before operation, just after operation, and 4 and 8 h after operation	↓ Respiratory chain failure; restoration of the redox status	[124]
CLP	Rat	Liver, kidney, lung, heart, diaphragm	Melatonin 10 mg/kg	i.p. 30 min before and 6 h after operation	↑ Level of GSH; ↓ MDA; ↓ tissue oxidative damage	[83]
LPS *E. coli*	Mouse	Heart	SS-31 5 mg/kg	i.p., 30 min after LPS	↓ ROS; restoration of myocardial damage; ↑ ATP; ↓ mRNA level of IL-6, IL-1β, and TNF-α; ↓ apoptosis;↑ SOD and GPx	[82]
LPS *E. coli*	Mouse	Liver, serum	XJB-5-131 2 μmol/kg	i.v. 1 h before LPS	↓ Hepatic iNOS expression, ↓ blood nitrite level	[119]
LPS *E. coli*	Mouse	Hippocampus	SS-31 5 mg/kg	LPS microinjection in the hippocampi SS-31 i.p. 30 min before LPS and then once daily for 3 days thereafter	↓ ROS, MDA, IL-6, and TNF-α; ↑ SOD; ↓ hippocampal cell apoptosis; ↑ BDNF expression and synaptic protein levels, maintenance of hippocampal neuron morphology; ↓ memory impairment	[125]
CLP	Mouse	Lung, kidney, liver	SS-31 5 mg/kg	i.p, immediately and 5 h after operation	↓ ROS, MDA, TNF-α, MPO activity, iNOS, and NF-κB p65; ↑ ATP; ↓ apoptosis, ↓ the histological damage; ↓ organ dysfunction, no result on mouse survival rate	[126]
CLP	Mouse	Hippocampus	SS-31 5 mg/kg	i.p, immediately after operation and once daily for 6 days thereafter	↓ ROS; ↓ NLRP3 and IL-1β; ↑ ATP; ↓ mitochondrial dysfunction; ↓ apoptosis; ↓ behavior and cognitive deficits; ↓ mortality rate	[127]
Live *E. coli* bacteria	Rat	Serum	M40401 (SOD mimetic) 0.25, 2.5, 25 μmol/kg/h	i.v. infusion 0.5 and 3 h after bacterial challenge	Maintenance of a normal mean arterial pressure; ↓ TNF-α and IL-1β; ↓ mortality	[128]

## Data Availability

Not applicable.

## Abbreviations

AMPK	5’ AMP-activated protein kinase
BAP31	B-cell receptor-associated protein 31
BDNF	Brain-derived neurotrophic factor
CLP	Cecal ligation puncture
COX2	Cyclo-oxygenase 2
DMM	Dimethyl malonate
GSH	Glutathione
GPx	Glutathione peroxidase
ETC	Electron transport chain
Fis1	Fission protein 1
IL-1β	Interleukin 1 beta
IL-18	Interleukin 18
iNOS	Inducible NO-synthase
IMM	Inner mitochondrial membrane
LPS	Lipopolysaccharide
MDA	Malondialdehyde
Mito-AOX	Mitochondria-targeted antioxidants
MMP	Membrane mitochondrial potential
MPO	Myeloperoxidase
mtDNA	Mitochondrial DNA
mtROS	Mitochondrial reactive oxygen species
NADH	Nicotinamide adenine dinucleotide
NLRP3	Inflammasome, NLR family pyrin domain containing receptor 3
NF-kB	Nuclear factor kappa B
Nrf2	Nuclear factor E2-related factor 2
OMM	Outer mitochondrial membrane
RET	Reverse electron transport
RNS	Reactive nitrogen species
ROS	Reactive oxygen species
TLR4	Toll-like receptor 4
TPP+	Triphenylphosphonium
SDH	Succinate dehydrogenase
SOD	Superoxide dismutase
